# Outbreak of avian mycobacteriosis in flocks of domestic pigeons: An epidemiological approach

**Published:** 2010-12

**Authors:** M Bolfion, M Salehi, J Ashrafi Helan, K Soleimani, R Keshavarz, R Aref Pajoohi, M Mohammad Taheri, K Tadayon, N Mosavari

**Affiliations:** 1 PPD Production Department, Razi Vaccine & Serum Research Institute, Karaj, Iran.; 2 North Tehran Branch, Islamic Azad University, Tehran, Iran.; 3 Veterinary School, University of Tabriz, Tabriz, Iran.

**Keywords:** *Mycobacterium avium* complex, pigeon, mycobacteriosis, PCR, epidemiology.

## Abstract

**Background and Objectives:**

Pigeons are extensively kept for homing and racing purposes in Iran. The main objective of this study was to investigate dissemination of *M. avium* subsp. a*vium* (MAA) in pigeon aviaries in Tabriz, North-western Iran.

**Materials and Methods:**

Postmortem pathologic specimens from thirty-nine out of 140 birds collected from private flocks (n=3), were subjected to bacterial culture out of which 3-4 mycobacterial isolates were recovered.

**Results:**

Applying a five-PCR diagnostic algorithm targeting short but definitive stretches of 16S rRNA and RV0577 genes, IS*6110*, IS*901* and IS*1245* genomic loci, proved all the isolates were MAA. They were either IS901+/IS1245+(n=22) or IS901+/IS1245- (n=12). When four healthy cattle sensitized against *Mycobacterium bovis* AN5 and *Mycobacterium avium* D4 were tuberculinated, the results confirmed the observed skin reactions against bovine tuberculin in animals sensitized with *M. avium* were large enough to complicate test interpretation.

**Conclusion:**

We believe the extent of such epidemiological impact deserves further investigation if progress in control of bovine tuberculosis is intended.

## INTRODUCTION

The Mycobacterium avium complex (MAC) consists of Mycobacterium avium subsp avium (MAA), Mycobacterium avium subsp silvaticum (MAS), Myco- bacterium avium subsp. paratuberculosis (MAP) and Mycobacterium avium subsp. hominissuis (MAH) plus Mycobacterium intracellulare (1, 2). These are the slow-growing, abundant bacteria in the environment that are all pathogens and cause serious diseases in birds and farm animals. Avian tuberculosis (AT) however, is often the consequence of infection of birds with Mycobacterium avium subsp avium (MAA), Mycobacterium avium subsp. silvaticum (3, 4) and Mycobacterium genavense (5–7).

With characterization of species-specific genomic markers and introduction of PCR and RFLP, it is now possible to differentiate MAC bacteria as IS*900* in MAP (8, 9), IS*901*/*902* in MAA as well as MAS (10, 4), IS*1245* in MAH and some strains of MAA (11, 12), IS*1642* in MAA (13) and finally FR300 in some MAA strains (10, 14) have been shown to be species- specific.

Avian tuberculosis has been reported in numerous species of pet, zoo, wild-life and avicultural birds (15, 7) including ring-neck doves (*Streptopelia risoria*) (7) and domestic pigeons (*Columbus livia domestica*) (16, 17).

The MAA bacterium is capable of developing disseminated disease in human individuals and such cases are now known to be larger than expected, an indication of epidemiological importance of this bacterium in the public health context (18). In addition, the MAA is able to infect cattle and develop hypersensitivity towards bovine tuberculin in the skin test, to form tuberculous lesions ultimately leading to decline of the economical value of carcass at abattoir, and finally to put other sensitive livestock and wildlife animals at risk of infection through discharge of the MAC isolate (19). In Iran we know very little about epidemiology of MAA in human and animals. In 1987, a study by the Iranian Veterinary Organization (IVO) showed MAA was frequently isolated from lymph nodes of cattle that were slaughtered following a positive result in the routine tuberculin test (IVO, unpublished data). Besides, there are anecdotal reports on isolation of MAC bacteria from domestic fowl in West Azerbaijan (Mosavari, unpublished data) and Fars (Tadayon, personal communications) provinces in north-west and central Iran respectively. Thus, the primary objective of this research was to isolate, identify and genetically characterize MAC bacteria collected from pathological lesions found in pigeon compatible with AT in Tabriz, where mycobacterial diseases both in human and animals have annual notifications.

## MATERIALS AND METHODS

Following complaints from three pigeon fanciers that many of their birds showed poor physical condition and unusual behavior for sometime e.g. cachexia, depression, no interest to fly, poor appetite and weight loss, these colonies were visited by the local veterinary officer and birds in their nests were closely examined. In consequence, 39 pigeons were collectively selected for postmortem examination on the grounds of their poor health condition. Birds were transferred to the diagnostic laboratory where, they were euthanized and necropsied. Microscopic slides were subsequently prepared from tissue impressions of the pathological lesions stained with Ziehl–Nielson technique in search for acid-fast bacilli (AFB). Using sterile material and equipments, all the associated specimens for each bird were pooled and grinded in a pestle and mortar containing sand. These included lesions found in liver, spleen, heart, gut, musculoskeletal system as well as gonads. The homogenized mixture was exposed to a cocktail of 5 ml N-acetyl-L-cysteine (5 g/l), 5 ml of sodium hydroxide (2 g/l) and 0.01 ml of sodium citrate solution for 15 min (20) in order to remove bacterial contaminators. About 5 ml of the supernatant was added to equal amount of phosphate buffer and centrifuged (3, 500 g/15 m). Again, 0.5 ml phosphate buffer was added to the deposit and the mixture was stirred to make the inoculation suspension. The inoculums were cultured on 4 culture slopes including glycerinated Lowenstein-Jensen (LJG) medium, pyruvate-enriched Lowenstein- Jensen medium (LJP), mycobactin J-supplemented Herrold-egg yolk medium and plain Herrold-egg yolk medium. The inoculated slopes were incubated at 37°C for 8 weeks.

**Molecular identification**. Each isolate was sub-cultured onto two fresh LJG slants in order to achieve bacterial growth enough for extraction of chromosomal DNA. Genomic DNA was extracted according to the Van Soolingen method (12). Concentration of extracted DNA was determined by gel electrophoresis and spectrophotometer. A PCR-based algorithm initially innovated in a different laboratory (21) was employed to enable differentiation of collected mycobacteria. This included five individual PCR assays targeting the 16S rRNA gene for identification of *Mycobacterium* spp. (22), Rv0577 gene as well as IS*6110* (specific for *Mycobacterium tuberculosis* complex) (22), IS*1245* (characteristic for MAC) (23, 10), and finally IS*901* (the identifier for MAA) (Table 1). PCRs were conducted as described elsewhere with incorporation of positive (*Mycobacterium bovis* AN5 and *Mycobacterium avium* subsp. *avium* D4 strains) and negative (double-distilled water) controls (10, 11, 23). Analysis of PCR amplicons was conducted on ethidium bromide-stained 2% agarose gels in a submerged electrophoresis system.

**Table 1:** The PCR-based diagnostic algorithm was employed by this study in order to identify the mycobacterial isolates collected from pigeons.

**Genomic marker**	**Holding species**	**PCR- product size**	**Primer sequences**	**References**
**16S rRNA**	*Mycobacteriun* spp.	543	1) CGG TGG GTA CTA GGT GTG GGT TTC	Huard et al. (2003)
			2) CTG CGA TTA CTA GCG ACT CCG ACT TCA	
**Rv 0577**	*M. tuberculosis* complex	786	1) ATG CCC AAG AGA AGC GAA TAC AGG CAA	Huard et al. (2003)
			2) CTA TTG CTG CGG TGC GGG CTT CAA	
**IS6110**	*M. tuberculosis* complex	245	1) CGT GAG GGC ATC GAG GTG GC	McHugh et al. (1997)
			2) GCG TAG GCG TCG GTG ACA AA	
**IS901**	*M. avium* subsp *avium*	1,108	1) GCA ACG GTT GTT GCT TGA AA	Kunze et al. (1991)
			2) TGA TAC GGC CGG AAT CGC GT	
**IS1245**	*M. avium* complex	427	1) AGG TGG CGT CGA GGA AGA C	Guerrero et al. (1995)
			2) GCC GCC GAA ACG ATC TAC	

**Sensitization/skin test experiments**. In the next phase, 4 healthy Holstein-Friesian cows in two two-animal groups were deep-intramuscularly administrated 0.5 ml fine-powdered heat-inactivated bacterial mass of *Mycobacterium bovis* AN5 and *Mycobacterium avium* subsp *avium* D4 strains respectively suspended in paraffin in order to immunologically sensitize them. Twelve weeks after initial injections, bovids were tuberculinated intradermally with 10,000 and 2,500 IU of bovine and avian PPD tuberculins respectively administrated in their left neck as instructed by the Iranian Veterinary Organization (IVO) in the national test-and-slaughter scheme. The experiment was further repeated for three times on three-cattle groups each included one bovid sensitized to *M. bovis*, one animal sensitized to *M. avium* and the last one non-sensitized as control.

## RESULTS

**Fig. 1:**
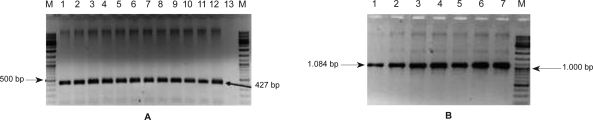
PCR amplification product of: **A)** the 427 bp specific fragment from IS*901*. Lanes 1–12, *Mycobacterium avium* subsp *avium* isolates colleted from pigeon in the study presented here**. Lanes M, DNA size marker with 100 bp rungs. **B)** the 1084 bp specific fragment from IS*1245*. Lanes 1–7, *Mycobacterium avium* subsp *avium* isolates colleted from pigeon in the study presented here**. Lane M, DNA size marker with 100 bp rungs.

In necropsy, characteristic AT granulomas were observed in internal organs, e.g. liver, spleen, heart, gut, kidney, ovaries, testes, eyes and musculoskeletal system specifically in legs, sternum and pectoral muscle of all the birds. Lesions in liver were particularly observable as 18 birds carried such lesions. In bacteriology, out of the 39 necropsied pigeons submitted for the test, 35 isolates were recovered all of which showed characteristic morphology of the MAC in bacterial culture and microscopy. The genomic material, however, was available from 34 isolates for molecular identification experiments. In 16S rRNA test, all the isolates produced a 543bp PCR fragment, an indication that they belonged to *Mycobacterium* genus (Table 1). None of the isolates in the study setting produced the 870bp amplicon in RV0577 assay confirming they were not member of *M. tuberculosis* complex (Table 1). In IS*901*-PCR experiment all the isolates produced an amplicon as large as 1,084 bp (Fig. 1B). This observation confirmed they carried the insertion sequence and therefore were MAA isolates. In IS*1245*-PCR experiment, 9 isolates produced the characteristic 427bp length amplicon (Fig. 1A).

In sensitization/skin test experiments, with no exception, all the sensitized cattle showed skin reactions towards bovine tuberculin although in those cattle sensitized to MAA D4 such reactions were overshadowed by the size of skin reactions against avian tuberculin. Nevertheless, they also produced a considerably large reaction to the bovine tuberculin.

## DISCUSSION

The present study explains naturally occurring mycobacteriosis of pigeons in Iran. There are a number of previous reports on infection of pigeons with MAA published elsewhere (1, 24, 17). To best of our knowledge, this is the first report on isolation of *M. avium* from pigeons in Iran. There is controversy on sensitivity of pigeons towards MAA, as some researches consider these birds highly susceptible (25, 24) while others reported them specifically resistant to both natural and experimental infection (26). In the study presented here, out of 140 birds bearing AT clinical demonstrations, 35 MAA isolates were recovered. This observation indicated sensitivity to infection. Whether the involved strains of MAA in this study show higher infectivity in the particular environment under study needs further investigation (27). Nevertheless, while all 34 collected isolates carried IS*901* element, a determinant of pathogenicity,22 genotyped isolates also carried the IS*1245* locus. Such isolates that belong to serotypes 1, 2 and 3 of MAA are considered as the most pathogenic strains of MAA in birds as well as humans (4, 28). Our observation that numerous gross tuberculous lesions were detected in internal organs of birds resembled the same postmortem scenario reported by other authors (29) but seems to conflict with findings of others that believe that columbiforms tubercles do not normally develop (30). This controversy might be explained by factors linked to the pigeon breed and also the strain pathogenicity.

Our sensitization test in cattle showed MAA had the potential to induce hypersensitivity reactions in the intradermal tuberculin test skin and affect the results interpretation. In 2007, some 50,000 bovids were tuberculinated in East Azerbaijan province where the present study conducted. This resulted in removing over 200 animals out of which 71 produced non-definitive test result. *Mycobacterium bovis* was apparently the main infecting agent of all these animals but no information is available on frequency of MAA infection in these bovids. Cattle are not normally sensitive to MAA but infection of digestive system in these animals via consumption of feedstuff has been previously reported (26). In an earlier comprehensive study by the Iranian Veterinary Organization (IVO), MAA isolates were isolated from lymph nodes of cattle with a positive test result. Pigeons are frequent visitors of cattle farms around their colonies where they search for food and water and also release their droppings thus infecting the environment and exposing cattle to pathogenic MAA.

In conclusion, we suggest further molecular-epidemiology studies in order to detect pathogenic strain of MAA in cattle and tracing the source(s) of MAA in the environment.
